# Supplementary material for: Digital Help for Substance Users (SU): A Systematic Review
Source: Int J Environ Res Public Health. 2022 Sep 8;19(18):11309. doi: 10.3390/ijerph191811309 (PMC9517354; doi:10.3390/ijerph191811309)
Supplement: Supplementary file 1 [file ijerph-19-11309-s001.zip › ijerph-1832629-supplementary.pdf]

SUPPLEMENTAL MATERIAL 1

SCOPUS search strategy
final search: 6

1	TITLE (digital OR computer OR software OR tablet OR app OR videogame OR seriousgame OR virtualreality)	825,580 results
2	(TITLE-ABS-KEY (mental AND health) AND TITLE-ABS-KEY (addiction OR dependence OR substance OR drug))	91,262 results
3	#1 and #2	403 results
4	#3 AND (LIMIT-TO (PUBYEAR , 2020) OR LIMIT-TO (PUBYEAR , 2019) OR LIMIT-TO (PUBYEAR , 2018) OR LIMIT-TO (PUBYEAR , 2017) OR LIMIT-TO (PUBYEAR , 2016) OR LIMIT-TO (PUBYEAR , 2015) OR LIMIT-TO (PUBYEAR , 2014) OR LIMIT-TO (PUBYEAR , 2013) OR LIMIT-TO (PUBYEAR , 2012) OR LIMIT-TO (PUBYEAR , 2011) OR LIMIT-TO (PUBYEAR , 2010))	198 results
5	#4 AND (LIMIT-TO (DOCTYPE , "ar")) AND (LIMIT-TO (LANGUAGE , "english") OR LIMIT-TO (LANGUAGE , "italian") OR LIMIT-TO (LANGUAGE , "spanish")) AND (LIMIT-TO (SRCTYPE , "j"))	135 results
6	#5 after manually removing review articles, comments and editorials	96 results

PsycInfo search strategy
Database - CINAHL Complete;APA PsycArticles;APA PsycInfo;Psychology and Behavioral Sciences Collection;American Bibliography of Slavic, East European, and Eurasian Studies;Dentistry & Oral Sciences Source;eBook Collection (EBSCOhost);RILM Abstracts of Music Literature
final search: s7

s1	TI digital OR computer OR software OR tablet OR app OR videogame OR seriousgame OR virtualreality	107,407 results
s2	AB mental AND health	415,190 results
s3	AB addiction OR dependence OR substance OR drug	765,313 results
s4	s1 and s2 and s3	504 results
s5	s4 from 2010 to 2020	306
s6	s5 only english	299
s7	s6 after manually removing review articles, book chapters, congress articles, comments and editorials	290

Web of Science search strategy
final search: #6

#1	digital OR computer OR software OR tablet OR app OR videogame OR seriousgame OR virtualreality (Title)	551,334 results
#2	AB=(mental AND health)	217,939 results
#3	AB=(addiction OR dependence OR substance OR drug)	2,554,591 results
#4	#1 and #2 and #3	140 results
#5	#1 and #2 and #3 and 2022 or 2021 or 2020 or 2019 or 2018 or 2017 or 2016 or 2015 or 2014 or 2012 or 2011 or 2010 (Publication Years) and English or Spanish (Languages)	133 results

#6	#5 after manually removing review articles, book chapters, congress articles, comments and editorials	100 results

PubMed search strategy
final search: #6

#1	digital[Title] OR computer[Title] OR software[Title] OR tablet[Title] OR app[Title] OR videogame[Title] OR seriousgame[Title] OR virtualreality[Title]	127,411 results
#2	mental[Title/Abstract] AND health[Title/Abstract]	243,306 results
#3	addiction[Title/Abstract] OR dependence[Title/Abstract] OR substance[Title/Abstract] OR drug[Title/Abstract]	1,757,837 results
#4	#1 and #2 and #3	131 results
#5	#1 and #2 and #3 Filters: English, Italian, Spanish, from 2010 - 2020	76 results
#6	#5 after manually removing review articles, book chapters, congress articles, comments and editorials	44 results

SUPPLEMENTAL MATERIAL 2

		Chander et al., 2021	Cucciare et al., 2021	Curtis et al., 2019	Danaher et al., 2019	de Ruijter et al., 2019	Drislane et al., 2020	Elison-Davies et al., 2020	Elison-Davies et al., 2021a	Elison-Davies et al., 2021b	Elison-Davies et al., 2017	Han et al., 2018	Kay-Lambkin et al., 2014	Leightley et al., 2018	Wernette et al., 2018	Wodarski et al., 2015	Qi et al., 2014	Zhang et al., 2019	Zhu et al., 2018
**sample**	*n of sample*	439	138	729	1271	269	780	5792	2571	1830	2311	75	35	31	50	5775	3309	30	40
	mean age	31	63,18	46,83	44,9	47,3		40,54	38,42	33,8	42,2	41,6	42,11		23,3			43,7	33,8
**primary substance**	*tobacco*				yes	yes		yes			yes						yes		
	alcohol	yes	yes	yes			yes	yes		yes	yes		yes	yes	yes	yes		yes	
	opioids			yes				yes	yes									yes	
	heroin			yes				yes			yes	yes							
	marijuana/cannabis			yes			yes	yes		yes	yes		yes		yes			yes	
	benzodiazepines			yes				yes											
	stimulants			yes				yes		yes	yes	yes						yes	yes
	others			yes				yes			yes								
	not specified substances															yes			
**gender**	*male*		133		280	6	344	3171	724	1064	1271	53	22	27				26	40
	female	439	8	725	991	263	436	2621	1847	753	1040	22	13	4	50			4	
	other			4						13									

**tools**	*PC/computer (on-line)/platform*	yes	yes	yes	yes	yes	yes	yes	yes	yes	yes		yes		yes	yes	yes		
	smartphone			yes	yes							yes		yes				yes	
	tablet			yes															yes
**approaches and models for digital treatments**	*BAI (Brief Alcohol Intervention)*	yes	yes																
	IVR (Interactive Voice Response)	yes																	
	D-RSS (digital recovery support services)			yes															
	informative intervention				yes											yes			
	e-learning					yes													
	CBI (computer guided theapy)						yes												
	CBT (Cognitive Behavioral Therapy)				yes			yes	yes	yes	yes	yes	yes						
	SDT (Self-Determination Therapy)											yes							
	HAPA (health action process approach) model													yes					
	IMB (Information-Motivation-Behavior) model														yes				
	ESCAPE (Effectiveness of computer-tailored Smoking Cessation Advice in Primary Care)																yes		
	Attentional Bias																	yes	yes
**outcomes**	*validated measures*	yes			yes	yes	yes	yes	yes	yes	yes	yes	yes	yes				yes	yes
	frequency of use	yes	yes		yes		yes	yes	yes			yes		yes	yes	yes	yes		
	abstinence			yes	yes	yes													
	recovery measures			yes				yes	yes	yes	yes								
	toxicology test	yes										yes			yes				
	follow-up	yes	yes	yes	yes	yes	yes	yes	yes	yes	yes	yes	yes	yes	yes	yes	yes		

**methodology and design**	*pre-post training*																	yes	yes
	subgroups/conditions	yes	yes	no	yes	yes	yes	no	no	no	no	yes	yes	no	yes	no	yes	no	yes
	randomized	yes	yes	no	yes	yes	yes	no	no	no	no	yes	no	no	yes	no	yes	no	yes
	control group/condition	yes	no	no	no	yes	yes	no	no	no	no	yes	no	no	yes	no	yes	no	yes
	placebo condition	no	no	no	no	no	no	no	no	no	no	no	no	no	no	no	no	no	no
**results**	*evidence of utility at least for one outcome*	yes	yes	yes	yes	yes	yes	yes	yes	yes	yes	yes	yes	yes	yes	yes	yes	yes	no
	evidence of utility comparing groups/conditions	yes	yes	no comparison	yes	yes	no	no comparison	no comparison	no comparison	no comparison	no comparison	no comparison	no comparison	yes	no comparison	no comparison	no comparison	no

		**Chander et al., 2021**	**Cucciare et al., 2021**	**Curtis et al., 2019**	**Danaher et al., 2019**	**de Ruijter et al., 2019**	**Drislane et al., 2020**	**Elison-Davies et al., 2020**	**Elison-Davies et al., 2021a**	**Elison-Davies et al., 2021b**	**Elison-Davies et al., 2017**	**Han et al., 2018**	**Kay-Lambkin et al., 2014**	**Leightley et al., 2018**	**Wernette et al., 2018**	**Wodarski et al., 2015**	**Wu et al., 2014**	**Zhang et al., 2019**	**Zhu et al., 2018**
**sample**	*n of sample*	439	138	729	1271	269	780	5792	2571	1830	2311	75	35	31	50	5775	3309	30	40
	mean age	31	63,18	46,83	44,9	47,3		40,54	38,42	33,8	42,2	41,6	42,11		23,3			43,7	33,8
**primary substance**	*tobacco*				yes	yes		yes			yes						yes		
	alcohol	yes	yes	yes			yes	yes		yes	yes		yes	yes	yes	yes		yes	
	opioids			yes				yes	yes									yes	
	heroin			yes				yes			yes	yes							
	marijuana/cannabis			yes			yes	yes		yes	yes		yes		yes			yes	
	benzodiazepines			yes				yes											
	stimulants			yes				yes		yes	yes	yes						yes	yes
	others			yes				yes			yes								
	not specified substances															yes			
**gender**	*male*		133		280	6	344	3171	724	1064	1271	53	22	27				26	40
	female	439	8	725	991	263	436	2621	1847	753	1040	22	13	4	50			4	
	other			4						13									
**tools**	*PC/computer (on-line)/platform*	yes	yes	yes	yes	yes	yes	yes	yes	yes	yes		yes		yes	yes	yes		
	smartphone			yes	yes							yes		yes				yes	
	tablet			yes															yes
**approaches and**	*BAI (Brief Alcohol Intervention)*	yes	yes																

**models for digital treatments**	*IVR (Interactive Voice Response)*	yes																	
	D-RSS (digital recovery support services)			yes															
	informative intervention				yes											yes			
	e-learning					yes													
	CBI (computer guided theapy)						yes												
	CBT (Cognitive Behavioral Therapy)				yes			yes	yes	yes	yes	yes	yes						
	SDT (Self-Determination Therapy)											yes							
	HAPA (health action process approach) model													yes					
	IMB (Information-Motivation-Behavior) model														yes				
	ESCAPE (Effectiveness of computer-tailored Smoking Cessation Advice in Primary Care)																yes		
	Attentional Bias																	yes	yes
**outcomes**	*validated measures*	yes			yes	yes	yes	yes	yes	yes	yes	yes	yes	yes				yes	yes
	frequency of use	yes	yes		yes		yes	yes	yes			yes		yes	yes	yes	yes		
	abstinence			yes	yes	yes													
	recovery measures			yes				yes	yes	yes	yes								
	toxicology test	yes										yes			yes				
**methodology and design**	*follow-up*	yes	yes	yes	yes	yes	yes	yes	yes	yes	yes	yes	yes	yes	yes	yes	yes		
	pre-post training																	yes	yes
	subgroups/conditions	yes	yes	no	yes	yes	yes	no	no	no	no	yes	yes	no	yes	no	yes	no	yes
	randomized	yes	yes	no	yes	yes	yes	no	no	no	no	yes	no	no	yes	no	yes	no	yes
	control group/condition	yes	no	no	no	yes	yes	no	no	no	no	yes	no	no	yes	no	yes	no	yes

	placebo condition	no	no	no	no	no	no	no	no	no	no	no	no	no	no	no	no	no	no
**results**	*evidence of utility at least for one outcome*	yes	yes	yes	yes	yes	yes	yes	yes	yes	yes	yes	yes	yes	yes	yes	yes	yes	no
	evidence of utility comparing groups/conditions	yes	yes	no comparison	yes	yes	no	no comparison	no comparison	no comparison	no comparison	no comparison	no comparison	no comparison	yes	no comparison	no comparison	no comparison	no
